# Extracellular vesicles of pathogenic fungi: biogenesis, immune regulation and therapeutic potential

**DOI:** 10.3389/fcimb.2026.1922490

**Published:** 2026-10-27

**Authors:** Jing Wu, Xinyao Qu, Longfei Yang, Xuerui Wang, Li Xu, Yu Zhang

**Affiliations:** 1 Department of Clinical Laboratory, The Second Hospital of Jilin University, Changchun, China; 2 Good Clinical Practice (GCP) Office, The Affiliated Hospital of Changchun University of Chinese Medicine, Changchun, China; 3 Jilin Provincial Key Laboratory on Molecular and Chemical Genetics, The Second Hospital of Jilin University, Changchun, China

**Keywords:** diagnosis, extracellular vesicles, fungi, host immunity, therapy

## Abstract

Extracellular vesicles (EVs) encapsulate a variety of biologically active molecules, such as proteins, lipids, and nucleic acids, and have been recognized as crucial mediators of intercellular communication. The production of EVs has been observed across numerous fungal species. Upon infection by pathogenic microorganisms, EVs contribute to the regulation of various pathophysiological processes and are instrumental in the activation and modulation of host immune responses through the transport of molecules derived from either the pathogen or the host. Nonetheless, several aspects of fungal EV biology remain inadequately understood, particularly regarding their potential applications as biomarkers for clinical diagnosis and as therapeutic targets for fungal infections. This review consolidates the current understanding of fungal EVs, with a particular focus on their biogenesis and secretion, their roles in host-pathogen interactions, and their potential applications as diagnostic and therapeutic tools for fungal infections. This includes their translational potential as diagnostic biomarkers, antifungal drug delivery systems, and fungal vaccine candidates. Additionally, the review explores emerging technologies such as single-vesicle analysis, multi-omics profiling, EV engineering, and synthetic and semisynthetic vesicular systems. The discussion aims to offer new perspectives and directions for the early prevention and treatment of microbial infections, while establishing a foundation for further fundamental research and the clinical translation of EV-based strategies.

## Introduction

### Status of diagnosis and treatment of invasive fungal infections

In recent years, the extensive use of broad-spectrum antibiotics, antineoplastic drugs, glucocorticoids, and immunosuppressants, along with invasive procedures and treatments such as indwelling catheters in body cavities, mechanical ventilation, and organ transplantation, has increased the risk of microbial infections in immunocompromised patients. The incidence and mortality of invasive fungal infections (IFIs) are increasing significantly, with more than 150 million people worldwide affected by severe fungal infections. The number of deaths related to fungal infections exceeds 1.6 million annually, which is more than three times that of malaria (Brown et al., 2012; Bongomin et al., 2017; Wheeler et al., 2017). At the end of 2019, patients with COVID-19 pneumonia caused by SARS-CoV-2 were also found to have fungal infections (Chen et al., 2020). The main pathogens of IFIs are *Candida*, *Cryptococcus*, *Aspergillus*, and *Pneumocystis*. Furthermore, the number of IFI cases caused by dimorphic fungi, such as *Histoplasma*, *Coccidioides*, *Blastomyces*, *Sporothrix*, and *Talaromyces marneffei*, and other filamentous fungi, such as *Scedosporium*, *Mucor*, *Rhizopus*, and *Fusarium*, has also increased in recent years (Skiada et al., 2017).

Despite the growing burden of IFIs, the therapeutic options remain limited. The major classes of antifungal drugs, including azoles, polyenes, and echinocandins, are associated with treatment failure due to limited activity against certain fungal pathogens, adverse effects, drug interactions, and the emergence of antifungal resistance (Nicola et al., 2019). These limitations underscore the need for novel therapeutic strategies with greater efficacy and safety. Conventional diagnostic approaches, including culture, microscopic examination, and serological testing, may have limited sensitivity or require lengthy processing times, thereby hindering the early, accurate identification and quantification of infections (Arvanitis et al., 2014). Nucleic acid-based diagnostic technologies, particularly polymerase chain reaction (PCR) and isothermal amplification, have emerged as promising approaches because of their high sensitivity, specificity, and relatively short detection time. However, the efficiency and quality of DNA extraction remain critical determinants of nucleic acid-based detection. The complex fungal cell wall can hinder efficient nucleic acid extraction, while contamination during sample processing may compromise diagnostic accuracy (Avni et al., 2011; Alanio and Bretagne, 2014). In addition to nucleic acid detection, protein mass spectrometry can also be used for microbial identification. Currently, many medical institutions at home and abroad have applied Matrix-assisted laser desorption/ionization time-of-flight mass spectrometry (MALDI-TOF MS) to fungal identification. It has been applied to fungal identification with advantages such as high sensitivity, high throughput, and low cost, and yeast identification accuracy has been close to 100% (Firacative et al., 2012; Quiles-Melero et al., 2012). However, many problems remain in identifying filamentous fungi. For example, it is difficult to extract thallus protein because mycelia, spores, and fruiting bodies interfere, and the amount of protein profile data is limited (Terrero-Salcedo and Powers-Fletcher, 2020). The continued rise in IFI incidence and mortality reflects the combined challenges associated with disease etiology, diagnosis, and treatment. Therefore, effective control of IFIs requires a deeper understanding of fungal pathogenic mechanisms, identification of novel therapeutic targets, and the development of rapid, accurate diagnostic techniques alongside safe, effective treatment strategies.

While numerous previous reviews have summarized the biological characteristics and immunological functions of fungal extracellular vesicles (EVs), they have predominantly focused on general descriptions of EV composition, classification, secretion pathways, host–pathogen interactions, cellular origins, experimental models for functional and compositional analyses, and production both *in vitro* and *in vivo*, as well as biomarkers for EVs (Rodrigues et al., 2025).

In contrast, this review adopts a more comprehensive and clinically relevant perspective by examining the formation and release of fungal EVs, their mediated interactions with the host immune system, and their potential applications in the diagnosis and treatment of fungal infections. This includes their potential as diagnostic markers, vehicles for antifungal drug delivery, and candidates for fungal vaccines.

This review offers a comprehensive synthesis of EVs produced by prominent human pathogenic fungi, including *Cryptococcus*, *Candida*, *Aspergillus*, and dimorphic fungi. It examines the current understanding of EV biogenesis and cargo composition, as well as the role of fungal EVs in immune modulation, fungal virulence, and biofilm-associated antifungal resistance. The review emphasizes the significance of small non-coding RNAs within fungal EVs and pathogen recognition receptor (PRR) material, elucidates the specific pathways through which host immune cells internalize fungal EVs, and explores the context-dependent effects of EV-mediated signaling. Furthermore, it addresses the heterogeneity of EV cargo under varying environmental and infectious conditions, alongside the potentially protective or pathogenic roles of EV activity during infection.

This review not only explores the biological functions of fungal EVs but also investigates their translational potential for clinical applications, such as their use as diagnostic biomarkers, vaccine platforms, drug delivery systems, and therapeutic targets. Additionally, it addresses emerging technologies, including single-vesicle analysis, multi-omics profiling, and EV engineering, along with synthetic and semi-synthetic vesicular systems, which are promising for advancing the characterization and clinical application of fungal EVs. By integrating mechanistic insights with novel translational strategies, the review seeks to elucidate the diverse roles of fungal EVs in infectious diseases and identify opportunities for their development as diagnostic and therapeutic tools.

### Formation and release of fungal extracellular vesicles

EVs are membranous structures that cells actively secrete. Because a universally accepted nomenclature based solely on EV biogenesis remains unavailable, the International Society for Extracellular Vesicles (ISEV) recommends using “extracellular vesicle” as a generic term and keyword for all secreted vesicles (Merchant et al., 2017). Based on their size, EVs can be broadly classified into three categories: apoptotic bodies (approximately 800–1000 nm in diameter), microvesicles (approximately 100–1000 nm in diameter), and exosomes (approximately 30–150 nm in diameter). Diverse cell types produce EVs, which contain a complex cargo of nucleic acids, lipids, proteins, and other small molecules. By transporting these biologically active components between cells, EVs mediate intercellular communication and contribute to a wide range of physiological and pathological processes, including immune regulation, infection, inflammation, and tumor development (Todorova et al., 2017; Van Niel et al., 2018; Wiklander et al., 2019; Möller and Lobb, 2020). Fungal EVs have attracted increasing attention because their cargo may contribute to fungal physiology, pathogenicity, and host–pathogen interactions. Among their diverse molecular components, small non-coding RNAs (sncRNAs), including miRNA-like molecules, tRNA fragments, small nuclear RNAs (snRNAs), and small nucleolar RNAs (snoRNAs), have emerged as an important area of investigation (Dallastella et al., 2023). Evidence indicates that fungal sncRNAs can be selectively packaged into EVs and delivered to recipient cells, where they may affect gene expression, immune responses, fungal growth, and other biological processes (Dallastella et al., 2023). The selective loading of specific sncRNAs in fungal EVs suggests that their cargo composition is regulated rather than being a random representation of intracellular RNA (He et al., 2021). In infection, fungi may transport virulence-associated sncRNAs to host immune cells *via* EVs, potentially altering host immune responses and facilitating fungal persistence (Pandey et al., 2020). The selective packaging of molecular cargo also supports the potential use of fungal EVs as biomarkers (Gandhi et al., 2022; Jnana et al., 2023; Ullah et al., 2023). Because the molecular composition of EVs can reflect the physiological or pathological state of their donor cells, analyzing EV-associated sncRNAs in biological fluids may provide non-invasive approaches for early detection, prognosis, and treatment monitoring of fungal infections. In addition, many studies have shown that EVs have strong potential as novel liquid biopsy markers (Rizzo et al., 2023; Yuan et al., 2024; de Rezende et al., 2025; Lee et al., 2025). Moreover, as endogenous substances with excellent intercellular transport, they are expected to serve as a natural carrier for a new generation of drug delivery. It has broad application prospects in the diagnosis, treatment, and prognostic assessment of various diseases (Merchant et al., 2017; Wiklander et al., 2019).

Research on fungal EVs remains relatively limited compared with that on mammalian EVs. Researchers first isolated and characterized fungal EVs from *Cryptococcus neoformans* in 2007. Since then, EVs have been reported in at least 19 other fungal species, including 12 yeast (phase) fungi, such as *Histoplasma capsulatum*, *Cryptococcus gattii*, *Candida albicans*, *Candida parapsilosis*, *Sporothrix schenckii*, *Sporothrix brasiliensis, Paracoccidioides brasiliensis*, *Paracoccidioides lutzii, Malassezia sympodialis*, *Saccharomyces cerevisiae, Pichia fermentans*, and *Exophiala dermatitidis* (Albuquerque et al., 2008; Gehrmann et al., 2011; Vallejo et al., 2011; Vargas et al., 2015; Bielska et al., 2018; Ikeda et al., 2018; Leone et al., 2018; Peres da Silva et al., 2019; Lavrin et al., 2020) and 7 species of filamentous fungi, such as *Trichoderma reesei (*de Paula et al., 2019), *Alternaria infectoria (*Silva et al., 2013), *Fusarium oxysporum* f.sp. *vasinfectum (*Bleackley et al., 2020), *Trichophyton interdigitale* (Bitencourt et al., 2018), *Rhizopus delemar (*Liu et al., 2018), *Aspergillus fumigatus* and *Aspergillus flavus* (Souza et al., 2019; Brauer et al., 2020; Rizzo et al., 2020).

In eukaryotic cells, EVs are generally formed through two major pathways. One involves outward membrane budding, producing EVs commonly called microvesicles, ectosomes, or microparticles, typically 100–1000 nm in size (Cocucci and Meldolesi, 2015). The other involves the endosomal pathway, in which multivesicular bodies (MVBs) form intraluminal vesicles. MVBs then fuse with the plasma membrane and release these vesicles into the extracellular environment as exosomes. This process can involve the endosomal sorting complex required for transport (ESCRT) machinery or ESCRT-independent mechanisms (Hanson and Cashikar, 2012). Studies have shown that MVB-like structures occur in *C. neoformans* and that *S. cerevisiae* with ESCRT mutations can still produce EVs, though their contents differ (Rodrigues et al., 2008; Oliveira et al., 2010a). In *C. albicans* biofilms, EVs contain ESCRT-associated proteins, including Hse1 and Vps27, providing further evidence for the involvement of endosomal trafficking machinery in fungal EV production (Zarnowski et al., 2018). Studies of *C. neoformans* have also identified distinct EV populations at the cell periphery, including vesicles of approximately 100 nm and 300 nm in diameter. The formation of microsomes is similar to that of exosomes, whereas macro EVs are produced by membrane budding, as with microvesicles (Takeo et al., 1973; Cocucci et al., 2009; Wolf et al., 2014).

The mechanisms underlying EV release by eukaryotic cells remain incompletely understood. Cytoskeletal components, including tubulin and actin filaments, molecular switches such as small GTPases, and membrane fusion machinery contribute to EV trafficking and release (Cai et al., 2007). However, fungal EV secretion has not been systematically characterized, and the relative contributions of different secretion pathways remain unclear. Current evidence suggests that fungi employ both conventional and unconventional secretory pathways for EV release (Panepinto et al., 2009; Oliveira et al., 2013; Bielska and May, 2019). In the conventional pathway, Sec6 has been implicated in EV secretion in *C. neoformans* (Panepinto et al., 2009), whereas Sec4, a Rab-family GTPase, regulates EV composition and release of EVs in *S. cerevisiae* (Oliveira et al., 2010a). In unconventional secretion, Golgi reassembly stacking proteins (GRASPs) have been implicated in EV release by *S. cerevisiae* and *C. neoformans* (Peres da Silva et al., 2015; Peres da Silva et al., 2018). A distinctive challenge in fungal EV biology is the cell wall, which maintains cell morphology and protects fungal cells against mechanical and environmental stress. The cell wall is also dynamically remodeled during growth, cell division, and stress exposure. Its presence initially led to the assumption that fungi could not release membrane-bound vesicles into the extracellular environment. Subsequent studies have established that fungal EVs can cross the cell wall, although the mechanisms responsible for this process remain unresolved (Lee et al., 2009; Rodrigues et al., 2014; Coelho and Casadevall, 2019). Proposed explanations include transient openings, localized cell-wall remodeling, wall elasticity, and turgor-driven passage (Rodrigues et al., 2014; Wolf et al., 2014). Walker et al. discussed a theoretical cell-wall pore-size estimate of approximately 5.8 nm and observed intact AmBisome liposomes traversing the cell walls of *C. albicans* and *C. neoformans* (Walker et al., 2018). These observations support wall deformability but do not directly establish how endogenous EVs are released. EV-associated glucanases and chitinases have been proposed to facilitate localized remodeling, potentially acting alongside wall elasticity and turgor pressure; however, their coordinated contribution to EV passage remains to be demonstrated (Rodrigues et al., 2014; Wolf et al., 2014). In encapsulated *Cryptococcus*, the polysaccharide capsule constitutes an additional potential barrier beyond the cell wall that EVs must traverse to reach the extracellular environment, although the mechanisms of capsular transit remain poorly understood (Mota et al., 2025). Rodrigues et al. (2014) reported that in the EVs produced by *C. albicans*, *P. brasiliensis*, *H. capsulatum*, *S. cerevisiae*, and *C. neoformans*, there were degrading enzymes of glucan and chitin, the main components of the fungal cell wall. *A. fumigatus* with and without cell walls produced different numbers and contents of EVs (Rizzo et al., 2020). These observations suggest a bidirectional relationship between EVs and the fungal cell wall, whereby EVs may contribute to cell-wall remodeling, while cell-wall architecture may, in turn, influence EV release and composition. However, the molecular mechanisms underlying this relationship remain poorly understood. Key unanswered questions include whether EVs directly participate in cell-wall assembly and remodeling and which EV-associated molecules mediate these processes (Bongomin et al., 2017).

In the canonical ESCRT-dependent pathway, ESCRT complexes coordinate endosomal membrane remodeling and the formation of intraluminal vesicles within multivesicular bodies (MVBs). Fusion of MVBs with the plasma membrane can subsequently release these vesicles into the extracellular environment (Brown et al., 2012; Tian et al., 2023). The extent to which this pathway contributes to EV production in different fungal species remains to be fully established. In *C. neoformans* and *C. albicans*, mutations in ESCRT-associated proteins were shown to alter EV cargo composition, virulence-associated molecule export, and vesicle-mediated immune modulation, underscoring the importance of the ESCRT machinery in fungal EV formation and cargo sorting. However, accumulating evidence suggests that fungal cells can also generate EVs through ESCRT-independent pathways (Liu and Hu, 2023). Studies in *S. cerevisiae* have demonstrated that EV production persists in ESCRT mutant strains, although vesicle size distribution, RNA cargo, and protein composition are significantly altered. These observations support alternative or compensatory pathways underlying EV biogenesis. Several mechanisms have been proposed to contribute to ESCRT-independent EV formation, including lipid-dependent membrane remodeling, ceramide-enriched membrane domains, Golgi-associated secretion, autophagy-related trafficking, and plasma membrane budding. Regulatory proteins implicated in these processes include GRASPs, Rab GTPases, Sec-family proteins, and lipid-trafficking regulators such as Sec14p (Li et al., 2026a).

Environmental conditions may further affect EV production and cargo composition. Oxidative stress, hypoxia, nutrient limitation, antifungal exposure, and host immune pressure have been reported to alter fungal EV profiles. For example, caspofungin treatment alters EV production and cargo composition in *Candida auris*, whereas nutritional conditions can affect the inflammatory properties of *Cryptococcus* EVs. These observations suggest that fungal EV production is dynamically regulated by environmental and host-associated stimuli and may contribute to fungal adaptation and pathogenicity (Ullah et al., 2023).

Despite these advances, several fundamental questions remain unresolved. Key issues include how specific cargo molecules are selectively sorted into distinct EV populations, how conventional and unconventional secretion pathways are coordinated, how EVs cross the fungal cell wall, and whether pathogenic fungi activate specialized EV biogenesis programs during host infection. Future research integrating high-resolution imaging, fungal genetics, lipidomics, and single-vesicle multi-omics may help clarify these mechanisms and further elucidate the contributions of EVs to fungal physiology, host–pathogen interactions, and disease progression (**Figure 1**).

**Figure 1:**
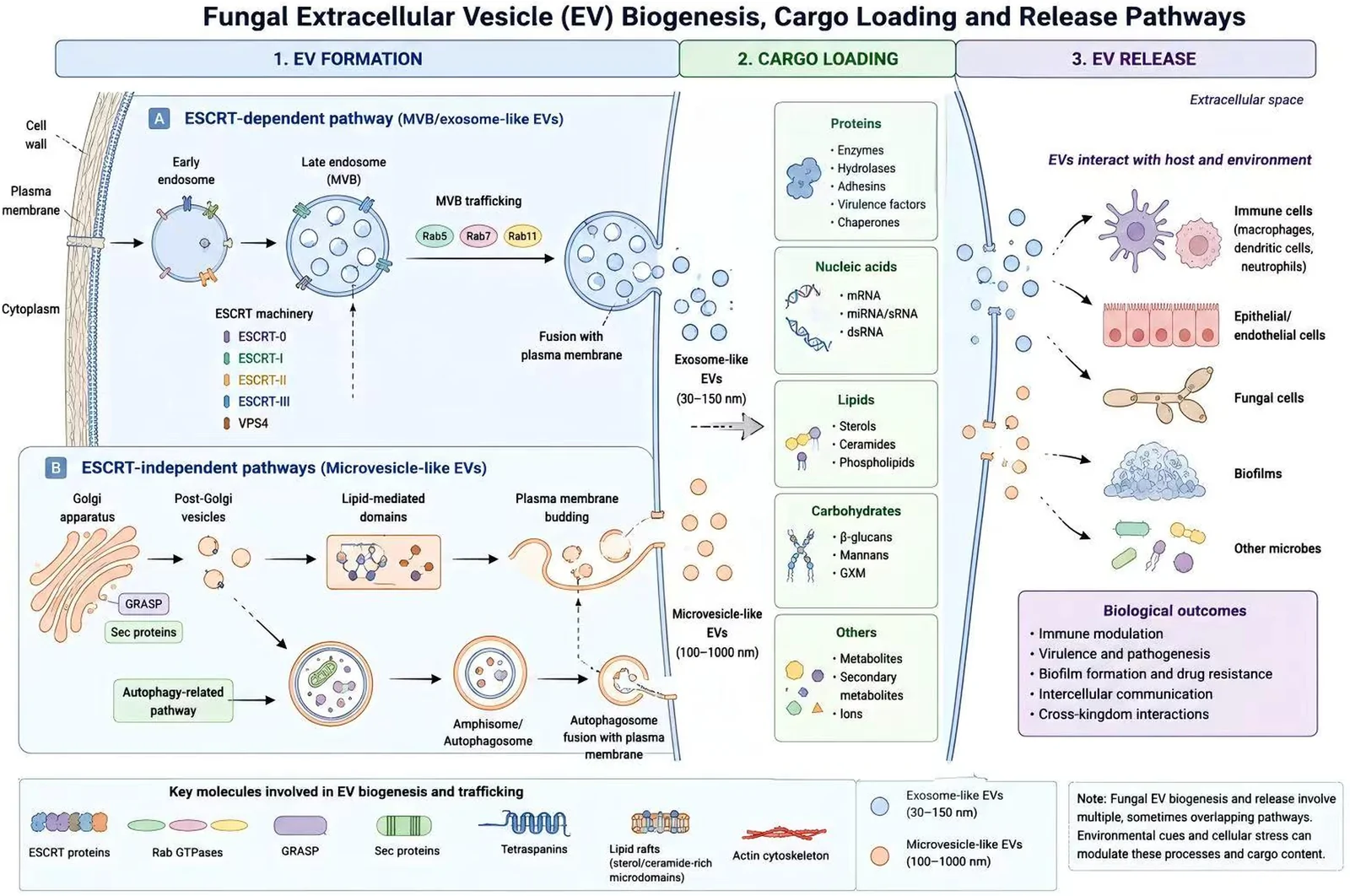
Proposed mechanisms of fungal extracellular vesicle (EV) biogenesis, cargo loading, and secretion pathways. Fungal EV biogenesis occurs through both ESCRT-dependent and ESCRT-independent pathways. In the ESCRT-dependent pathway, endosomal membrane invagination forms intraluminal vesicles within multivesicular bodies (MVBs), which then fuse with the plasma membrane and release exosome-like EVs. This process involves ESCRT complexes (ESCRT-0, -I, -II, and -III), VPS proteins, Rab GTPases, and membrane trafficking regulators. In the ESCRT-independent pathway, EVs are generated through plasma membrane budding, lipid raft-associated membrane remodeling, Golgi-derived secretory pathways, and autophagy-related vesicle trafficking, resulting in the release of microvesicle-like EVs. Fungal EVs contain diverse bioactive cargo, including proteins (enzymes, adhesins, virulence factors, and chaperones), nucleic acids (mRNA, small RNAs, and dsRNA), lipids (sterols, ceramides, and phospholipids), carbohydrates (β-glucans, mannans, and GXM), metabolites, and other signaling molecules. Environmental stress, nutrient availability, antifungal exposure, and host-derived signals may regulate EV cargo composition and secretion. Following release, fungal EVs can interact with host immune cells, epithelial cells, fungal biofilms, and microbial communities, thereby influencing immune responses, fungal virulence, intercellular communication, biofilm formation, antifungal resistance, and host–pathogen interactions. The schematic diagram was created using BioRender (https://www.biorender.com/).

### EV-mediated interaction between fungi and the host immune system

Fungal EVs facilitate pathogen–host communication, and recipient host cells must internalize their bioactive cargo to induce intracellular or immunomodulatory effects. EV uptake occurs through multiple mechanisms, including clathrin-mediated endocytosis (CME), caveolin-dependent endocytosis, macropinocytosis, and direct membrane fusion. The relative contribution of these pathways depends on EV properties, such as size, surface charge, and ligand composition, as well as the recipient cell type and its activation state. CME is among the best-characterized mechanisms of EV uptake. For example, small RNAs transported by *Botrytis cinerea* EVs can enter *Arabidopsis thaliana* cells through CME and subsequently regulate immune-related genes (He et al., 2023). Similarly, inhibition of CME reduces uptake of *C. albicans* EVs by macrophages, suggesting that this pathway contributes to EV internalization and subsequent immune responses (Kwaku et al., 2025). Caveolin-dependent uptake involves lipid-rich membrane microdomains and may represent an alternative pathway for EV entry, although its specific role in fungal EV internalization remains poorly understood (Souza et al., 2021). Macropinocytosis is an actin-dependent process that facilitates the non-selective uptake of extracellular materials and may be particularly important for the internalization of larger or aggregated EVs.

Differences in EV uptake efficiency across fungal species have been associated, at least in part, with differences in their surface components (Kwaku et al., 2025). In macrophages, *C. albicans* EV-induced inflammatory responses have also been associated with increased macropinocytic activity (Piraine et al., 2026). Direct membrane fusion represents another potential mechanism of EV entry and occurs independently of clathrin- or caveolin-mediated endocytosis. Membrane lipids and fusion-associated proteins may influence this process. Tetraspanins identified in fungal EVs may facilitate EV adhesion and membrane interactions; however, their direct involvement in fungal EV–host cell fusion remains to be established (Wang et al., 2025a). It is important to note that this evidence is primarily derived from studies on plant pathogens and thus should not be generalized without further investigation. In *C. neoformans*, increased phosphatidylserine exposure on EVs was not associated with recognition by classical phosphatidylserine receptors, suggesting that alternative mechanisms may contribute to EV uptake (Pawar et al., 2026). Overall, fungal EV internalization is not mediated by a single pathway but depends on the combined properties of the vesicles and recipient cells. Macrophages may preferentially utilize actin-dependent uptake mechanisms, whereas dendritic cells can efficiently internalize EVs through CME and may subsequently process their cargo for antigen presentation (Kwaku et al., 2024). EV surface composition also affects uptake efficiency; differences among EVs from *C. albicans*, *S. cerevisiae*, *C. neoformans*, and *A. fumigatus* have been associated with distinct internalization profiles (Kwaku et al., 2025). In some fungi, surface-associated polysaccharides or hydrophobins may reduce macrophage uptake and thereby influence immune recognition (Kwaku et al., 2025). Furthermore, interactions between EV-associated ligands and host pattern-recognition receptors (PRRs), including Dectin-1 and Toll-like receptors (TLRs), can affect both EV uptake and downstream signaling (Honorato et al., 2024). Thus, fungal EV internalization is a dynamic, multifactorial process that underpins the complexity of their immunomodulatory functions.

Following internalization or surface recognition, fungal EVs can activate host innate immune responses through pathogen-associated molecular patterns (PAMPs) present on or within the vesicles. These components include β-glucans, mannans, chitin-derived molecules, glycolipids, and other fungal factors that are recognized by host PRRs (Kwaku et al., 2025; Wang et al., 2025b). Variation in the abundance and accessibility of these PAMPs among fungal EVs can produce distinct patterns of PRR activation and inflammatory responses. Dectin-1 recognizes exposed β-1,3-glucans and activates the ITAM–Syk–CARD9 pathway, which subsequently triggers downstream NF-κB and MAPK signaling (Iliev et al., 2021; Honorato et al., 2024; Shi et al., 2025; Ameri Shah Reza et al., 2026). TLR2 can recognize fungal lipids and glycoproteins associated with EVs, although surface polysaccharides and hydrophobins may alter receptor accessibility and EV uptake (Kwaku et al., 2025). TLR2 activation generally signals through the MyD88 pathway and can induce cytokine responses that vary by fungal EV source (Marks et al., 2021; Piraine et al., 2026). TLR4 has also been implicated in recognizing fungal EV-associated components, including mannan (depending on O- and N-linked modifications) (Honorato et al., 2024) and cryptococcal glucuronoxylomannan (GXM) (Sato and Kawakami, 2022). TLR4 signaling can proceed through both MyD88-dependent and TRIF-dependent pathways, leading to inflammatory responses and type I interferon production (Choudhary et al., 2026). Importantly, fungal EVs can engage multiple PRRs simultaneously. Cooperative signaling between Dectin-1 and TLR4, for example, can affect dendritic-cell activation (Honorato et al., 2024), while interactions between CR3 and TLR4 have been implicated in regulating EV release (Halder et al., 2022). Such receptor-level and downstream signaling interactions allow host cells to integrate multiple fungal signals. They may determine whether the resulting response is predominantly pro-inflammatory, anti-inflammatory, or immunoregulatory, thus affecting infection outcomes.

Recent studies have further shown that the immunomodulatory effects of fungal EVs are closely linked to their specific bioactive cargo and the downstream host signaling pathways they activate. For example, GXM-enriched EVs from *C. neoformans* suppress Th1-type immune responses by enhancing IL-10 production and inhibiting IL-12 secretion, thereby modulating signaling pathways in macrophages and monocytes. In comparison, phospholipid-rich EVs from *C. albicans* activate NF-κB signaling in macrophages and dendritic cells, promoting the production of pro-inflammatory mediators, including tumor necrosis factor-α (TNF-α), IL-6, and IL-12 (Nenciarini and Cavalieri, 2023).

In *A. fumigatus*, EV-associated proteins and polysaccharides activate macrophages and neutrophils through pathways linked to inflammasome activation and reactive oxygen species (ROS) production, increasing IL-1β and other inflammatory mediators. Similarly, EVs derived from *P. brasiliensis* promote M1 macrophage polarization and inflammasome activation, increasing IL-1β production and amplifying antifungal inflammatory responses (Freitas et al., 2023). In addition to proteins and polysaccharides, fungal EV-associated RNAs, lipids, heat shock proteins, and virulence-associated enzymes may regulate host immunity through TLR signaling, MAPK activation, oxidative stress responses, and changes in macrophage metabolism (Li et al., 2026b). These findings indicate that fungal EVs are not simply passive carriers of individual virulence factors but complex signaling platforms that can coordinate multiple host responses during infection. Fungal cells can both release and take up EVs: uptake by recipient cells has been demonstrated in *C. albicans*, *A. fumigatus*, and *P. brasiliensis*, with associated changes in gene expression and cellular phenotypes (Bitencourt et al., 2022). Together, these findings support bidirectional EV-mediated communication, although the mechanisms governing inward and outward passage across fungal cell walls remain incompletely understood.

Despite substantial differences among fungal species, EVs from various pathogenic fungi induce several common patterns of innate immune activation. Fungal EVs can stimulate macrophages, dendritic cells, and neutrophils, promoting the production of inflammatory mediators such as TNF-α, IL-1β, IL-6, IL-12, and nitric oxide (NO). These immune responses may enhance phagocytosis, influence macrophage polarization, and promote the activation of antifungal defense mechanisms (Lai et al., 2023).

Host immunity is essential for controlling invasive fungal infections and involves coordinated innate and adaptive responses (Drummond and Lionakis, 2019). Fungal EVs are immunogenic and can significantly modulate these responses, particularly during the early stages of host–pathogen interaction (Patin et al., 2019). EVs have been characterized in major human fungal pathogens, including *Cryptococcus*, *Candida*, and *Aspergillus*, as well as in several dimorphic fungi. The following sections discuss pathogen-specific EV-mediated interactions with the host immune system, emphasizing how fungal EV cargo affects immune recognition, inflammatory responses, immune evasion, and disease progression.

### Cryptococcus

*C. neoformans* and *C. gattii* are the major pathogenic species responsible for cryptococcosis. *C. neoformans* primarily causes disease in individuals with impaired cell-mediated immunity, particularly those with advanced HIV infection, whereas *C. gattii* more frequently causes disease in immunocompetent or otherwise apparently healthy individuals. Several pathogenic *Cryptococcus* species can cause invasive pulmonary disease and central nervous system infection, with cryptococcal meningitis and meningoencephalitis representing major clinical manifestations that account for more than 80% of cases and have a high fatality rate (Hagen et al., 2015; Iyer et al., 2021). The global burden of cryptococcosis remains substantial, particularly among people with advanced HIV infection, and mortality remains high despite the availability of antifungal therapy. Statistics show that there are nearly one million new cases of cryptococcosis in the world every year, and more than 600,000 people die even after 3 months of treatment (Park et al., 2009). *Cryptococcus* pathogenicity is strongly associated with its cell wall polysaccharide capsule, which limits phagocytosis and interferes with host immune clearance. GXM is the major capsule component, accounting for about 90% of its polysaccharide content, with smaller amounts of g galactose, xylosin, mannan, and mannan protein. GXM is also an important component of *C. neoformans* EVs and contributes to their immunomodulatory activity. EV-associated GXM can suppress immune responses in monocytes, macrophages, neutrophils, and T cells (Monari et al., 2006; Rodrigues et al., 2008). In monocytes, GXM-containing EVs promote IL-10 production and reduce IL-12 secretion, thereby suppressing the development of a Th1-type immune response (Retini et al., 2001). GXM-containing EVs have also been associated with macrophage cytotoxicity by activating the Fas/FasL pathway in both *in vivo* and *in vitro* models (Monari et al., 2005; Villena et al., 2008). These findings indicate that *C. neoformans* EVs can transport capsule-associated immunomodulatory molecules beyond the fungal cell surface and thereby regulate host immune responses at sites distant from the fungal cell.

However, *C. neoformans* EVs are not exclusively immunosuppressive. EV exposure can induce macrophages to produce both regulatory cytokines, including TGF-β and IL-10, and pro-inflammatory mediators such as TNF-α and NO. In some experimental settings, EV stimulation also increases macrophage phagocytic and fungicidal activity (Oliveira et al., 2010b). These findings suggest that the biological effects of *Cryptococcus* EVs depend on EV composition and the physiological conditions under which they are produced. Marina et al. (2020) reported that EVs produced by *C. neoformans* under nutrient-rich conditions elicited weaker inflammatory responses than EVs produced under nutrient-limited conditions. EVs derived from acapsular ΔCap67 cells also induced stronger inflammatory responses than those from encapsulated cells, consistent with previous findings that the capsule can influence EV-mediated immune activation (Oliveira et al., 2010b; Marina et al., 2020).

Furthermore, *C. neoformans* EVs can provide an early stimulus for IL-1β production, indicating that they may participate in inflammasome-associated inflammatory responses (Oliveira et al., 2010b). EV-mediated immunomodulation is also observed in *C. gattii*. EVs from virulent strains can alter macrophage responses and promote intracellular proliferation of attenuated *Cryptococcus* strains, suggesting that EVs may contribute to infection establishment and maintenance (Bielska et al., 2018). Beyond proteins and polysaccharides, small molecules constitute another component of *Cryptococcus* EV cargo. Reis et al. characterized small molecules from EVs produced by *Cryptococcus deuterogattii* under solid-culture conditions using chromatography and spectroscopy. They identified a peptide associated with increased survival of *Galleria mellonella* following infection (Reis et al., 2021). Castelli et al. further identified NOP16-associated components within the small-molecule fraction of *C. deuterogattii* EVs. Deletion of *NOP16* reduced EV production and altered their small-molecule composition, suggesting that this gene contributes to EV biogenesis and cargo organization and may thereby influence *Cryptococcus* virulence (Castelli et al., 2022). Rizzo et al. reported that prophylactic administration of *C. neoformans* EVs induced antibody responses and prolonged survival in infected animals (Rizzo et al., 2021). Structural and compositional analyses further identified known protective antigens on the EV surface. The particulate nature of EVs and their ability to present multiple fungal antigens make them attractive candidates for vaccine development (Rizzo et al., 2021).

### Candida

*C. albicans* is a common commensal fungus of humans and is frequently found in the oral cavity, gastrointestinal tract, and vagina. Although it generally exists without causing disease in healthy individuals, disrupted host immunity or barrier integrity can lead to superficial infections, candidemia, and disseminated candidiasis (Kim and Sudbery, 2011). Increasing evidence indicates that EVs contribute to *Candida* adaptation, host–pathogen interactions, immune modulation, and virulence.

*C. albicans* EVs can affect both fungal physiology and host immune responses. EVs have been shown to activate the L-arginine/NO pathway and promote fungal growth, accompanied by increased NO production and upregulation of the NO dioxygenase gene *YHB1*. Increased Yhb1 activity may reduce intracellular ROS accumulation and apoptosis, thereby supporting fungal survival (Wei et al., 2023). EVs also contain the high-affinity glucose transporter Hgt1p, which can bind complement factor H and interfere with alternative complement pathway activation. Blocking Hgt1p increases neutrophil phagocytosis and intracellular killing of *C. albicans*, suggesting that EV-associated Hgt1p contributes to immune evasion (Kenno et al., 2019). Under the action of EVs of *C. albicans*, both the mouse macrophage cell line RAW264.7 and bone marrow-derived macrophages (BMDM) can produce NO, IL-12p40, and IL-10. The mouse macrophage cell line RAW264.7 and BMDM produce TGF-β and TNF-α, respectively. *C. albicans* EVs can also promote the maturation of bone marrow-derived dendritic cells (BMDCs), upregulate MHC-II and CD86 expression, and produce IL-12p40, TNF-α, and TGF-β (Vargas et al., 2015). The phospholipid component of *C. albicans* EVs plays an important role in activating immune cells and is associated with NF-κB pathway activation in BMDM and the macrophage cell line J774.14 (Wolf et al., 2015). During host cell infections, *C. albicans* EVs synergistically enhanced *C. albicans’s* destructive effects on host cells, including RAW264.7, HOK, TR146, and HGEC, suggesting that EV-mediated growth promotion enhanced *C. albicans* pathogenesi*s* (Wei et al., 2023). Recent studies have shown that *C. albicans* EVs inoculated with adjuvants stimulate the production of inflammatory mediators (IL-12p70, TNFα, and IFNγ) in immunosuppressed mice, reduce fungal burden, and promote antibody production. The researchers believe that fungal EVs may be a safe source of antigens for vaccine formulations (Vargas et al., 2020). Similarly, EVs from *C. auris* also increased the expression of MHC II, CD80, and CD86 on BMDCs (Zamith-Miranda et al., 2021). First-line therapeutic agents, such as caspofungin, can alter EVs secreted by *C. auris*, enabling the strain to evade treatment and persist in the host (Amatuzzi et al., 2022).

Proteomic analyses have identified proteins associated with immunogenicity and drug resistance in *C. auris* EVs, as well as abundant proteins related to *C. albicans*, including Mp65-like and Xog1-like proteins. These components have been proposed to reduce drug bioavailability through different mechanisms, helping the fungus survive in the presence of amphotericin B (Chan et al., 2022). EVs are also closely associated with *Candida* biofilm formation and antifungal resistance. *C. albicans* biofilms release EVs that contribute to extracellular matrix formation and can promote the resistance of biofilm communities to antifungal agents (Zarnowski et al., 2021; Honorato et al., 2022). EV cargo may exert cooperative or competitive effects among *Candida* species depending on the composition and requirements of the microbial community (Zarnowski et al., 2022a). In mixed-species biofilms, EVs can further improve the development and stability of both mono-species and polymicrobial biofilm communities (Zarnowski et al., 2022b). EVs in biofilm-associated drug resistance offer a potential therapeutic target. Turbinmicin, a recently described antifungal compound, has been reported to disrupt EV delivery during *C. albicans* biofilm development, impairing extracellular matrix assembly and rendering biofilm communities more susceptible to fungal killing (Zhang et al., 2020; Zhao et al., 2021). The ability of exogenous biofilm-derived EVs to restore the phenotype further supports the role of EV-mediated delivery in matrix formation and biofilm-associated resistance. These findings support targeting EV biogenesis or trafficking as an alternative strategy for treating fungal biofilms. EVs released by non-*albicans Candida* species may similarly contribute to biofilm development and infection pathogenesis (Kulig et al., 2022).

Since the yeast-to-hyphal transition is a key virulence feature of invasive candidiasis, fungal EVs can serve as signaling compartments and directly affect morphogenesis. Honorato et al. compared EVs secreted by *C. albicans* mycelial cells (HEV) and yeast EVs (YEVs) (Honorato et al., 2022) and found that HEV had cytotoxic effects on human macrophages and could induce TNF-α release, suggesting more pronounced effects on human immune cells (Martínez-López et al., 2022).

### Aspergillus

*Aspergillus* species are opportunistic fungi that can cause disease primarily in individuals with impaired immunity. Among the pathogenic species, *A. fumigatus* is the most common, followed by *A. flavus*, *Aspergillus niger*, and *Aspergillus terreus*. Aspergillosis encompasses allergic, chronic, and invasive forms, with invasive aspergillosis (IA) representing the most severe manifestation. Despite advances in diagnostic approaches and antifungal therapy, early diagnosis of IA remains challenging, and mortality is still high (50%-95%) in severely immunocompromised patients (Dagenais and Keller, 2009). Evidence for vesicle-like structures in *Aspergillus* dates back to ultrastructural observations by Gibson and Peberdy in 1972, who described granular structures resembling EVs in protoplasts of *Aspergillus nidulans* and called them subprotoplasts (Gibson and Peberdy, 1972). More direct characterization of EVs from pathogenic *Aspergillus* species was reported only recently, with EVs reported in *A. fumigatus* and *A. flavus* (Brauer et al., 2020; Rizzo et al., 2020). *A. fumigatus* EVs can be internalized by macrophages and stimulate the production of TNF-α and CCL2 while increasing macrophage phagocytic activity. Exposure of bone marrow-derived neutrophils to *A. fumigatus* EVs similarly increases phagocytic activity and induces production of inflammatory mediators, including TNF-α and IL-1β (Souza et al., 2019). Sec14p, a phospholipid transfer protein involved in the trafficking of phosphatidylinositol and phosphatidylcholine, contributes to vesicular transport in fungi (Bankaitis et al., 1990; Curwin et al., 2009). Turbinmicin targets the Sec14p-associated vesicle trafficking pathway and has been reported to significantly reduce EV production in *A. fumigatus* biofilms (Zhang et al., 2020). In animal models, turbinmicin demonstrated antifungal activity against *Candida* and *Aspergillus* species, supporting the potential of vesicle trafficking pathways as therapeutic targets (Zhao et al., 2021).

Researchers have also investigated the immunogenicity of *A. fumigatus* EVs as a potential basis for vaccine development. Mice pre-exposed to *A. fumigatus* EVs developed fungus-specific serum IgG responses. EV immunization reduced neutrophil infiltration into the alveolar space, protein extravasation, and production of inflammatory mediators, including IL-1β, IL-6, and CXCL1. Immunized animals also showed reduced pulmonary tissue injury, increased phagocytic activity, and improved fungal clearance (Souza et al., 2022). Importantly, combined immunization with *A. fumigatus* EVs and low-dose amphotericin B protected approximately 50% of infected animals in the experimental model (Souza et al., 2022). Bitencourt et al. found that EVs from a regular or irradiated culture triggered a stress response in fresh *A. fumigatus* cultures (Bitencourt et al., 2022). In addition to colony reduction formation, the mpkC gene is up-regulated in response to stress. Fungitoxic compounds in *A. fumigatus* EVs may cause these effect*s*. EVs of *A. flavus* can also induce the production of NO, TNF-α, IL-6, and IL-1β in mouse BMDM *in vitro*, promote their polarization to M1 macrophages, and enhance their phagocytic and killing abilities.

### Dimorphic fungi

Dimorphic fungi are a distinct group of pathogenic fungi that undergo morphological transitions in response to temperature. They generally grow in a filamentous or hyphal form at room temperature (approximately 25 °C) and transition to a pathogenic yeast or yeast-like form under mammalian host conditions, typically at 37 °C. This thermal dimorphism contributes to their ability to cause infections in both immunocompromised and immunocompetent individuals. Representative species include *H. capsulatum*, *P. brasiliensis*, *Blastomyces dermatitidis*, *Coccidioides immitis*, *T. marneffei*, and *S. schenckii* (Klein and Tebbets, 2007). Except for *S. schenckii*, which has a broad geographic distribution, infections caused by these fungi are generally endemic to specific regions. For example, histoplasmosis caused by *H. capsulatum* is prevalent in North and South America, whereas *P. brasiliensis* and *P. lutzii* are the major causative agents of paracoccidioidomycosis in Latin America (Van Dyke et al., 2019). *T. marneffei* infection is predominantly reported in China and Southeast Asia (Tam et al., 2015). Dimorphic fungal infections are estimated to affect approximately 10 million people worldwide (Fernandes et al., 2018). *S. schenckii*, the major causative agent of sporotrichosis, has a broader global distribution and is also prevalent in China, where the incidence of sporotrichosis has increased significantly (Sizar and Talati, 2023).

Rizzo et al. highlighted that Th1-type immunity is generally associated with effective infection control, whereas Th2/Th9-associated responses have been linked to disease progression and dysregulated inflammation (Rodríguez Molina and Tobón Orozco, 2018). However, these associations do not imply that pro-inflammatory responses are invariably protective. EVs from *P. brasiliensis* promote M1-like macrophage polarization, shift M2-like macrophages toward an M1-like phenotype, and activate inflammasome-related responses with increased IL-1β production *in vitro (*Da Silva et al., 2016). These findings demonstrate immune activation, but its protective significance requires assessment *in vivo*. Immunization with EVs and an adjuvant reduced pulmonary fungal burden in infected mice, enhanced activated T-cell and NKT-cell mobilization and pro-inflammatory cytokine production, and limited histological damage (Baltazar et al., 2021). In contrast, Octaviano et al. found that EVs from a virulent strain restored the ability of the non-virulent aPb18 strain to grow under oxidative and nitrosative stress, indicating that EVs can also enhance fungal stress resistance (Octaviano et al., 2022). Moreover, EVs from the attenuated variant (aEVs) induced inflammatory mediators in macrophages and mice. Pretreatment with aEVs exacerbated infection, increasing pulmonary fungal burden alongside TNF-α, IFN-γ, IL-6, and MCP-1 levels. Thus, increased pro-inflammatory mediator levels did not necessarily correspond to improved fungal control. Together, these findings suggest that the effects of EVs depend on their source and the exposure context, with potentially distinct consequences for host immunity and fungal fitness. A similar distinction between immune activation and protection is evident for *S. brasiliensis*. EVs from this fungus have been reported to carry virulence factors, enhance dendritic cell phagocytosis, and increase IL-12 and TNF-α production *in vitro*. Nevertheless, EV administration before subcutaneous infection increased fungal burden in BALB/c mice (Ikeda et al., 2018). These findings indicate that enhanced phagocytosis and cytokine production *in vitro* do not necessarily translate into protection *in vivo*. The overall outcome may reflect both the biological effects of EV cargo and the host response under the specific exposure conditions. Campos et al. co-cultured J774 macrophages with different concentrations of EVs, which promoted macrophage bactericidal activity, elevated IL-12 and IL-6 levels, and up-regulated MHC-II and CD86 expression, indicating that EVs play a protective role during *S. brasiliensis* infection (Campos et al., 2021). Incubation of EVs from *T. marneffei* yeast with macrophages has been reported to increase the expression of reactive oxygen species, nitric oxide, and several inflammatory factors, including IL-1β, IL-6, IL-10, and tumor necrosis factor (Yang et al., 2021). In addition, the expression of co-stimulatory molecules (CD80, CD86, and MHC-II) was also increased in EV-stimulated macrophages.

EVs from another important dimorphic fungus, *Malassezia*, have also been characterized. *Malassezia*-derived allergen-containing EVs induce high levels of the pro-inflammatory cytokine TNF-α and the anti-inflammatory cytokine IL-4, suggesting that these vesicles may exert dual immunomodulatory effects in atopic eczema (AE) (Gehrmann et al., 2011). Functional studies demonstrated that human keratinocytes and monocytes actively bind and internalize MalaEx derived from *M. sympodialis*. Given their high allergen content and ability to interact with skin cells, MalaEx may contribute to sensitization and the persistent cutaneous inflammation in AE (Johansson et al., 2018). Subsequent studies showed that MalaEx-induced keratinocyte activation and increased ICAM-1 expression may contribute to cutaneous defense against *M. sympodialis (*Vallhov et al., 2020). *Malassezia furfur* also releases EVs that keratinocytes can internalize, stimulating IL-6 production through an NF-κB-dependent pathway. These findings further clarify the role of EV-mediated signaling in *Malassezia* pathogenesis and may inform the development of therapeutic strategies (Zhang et al., 2019).

Studies of fungal EV cargo across species have identified several recurring features. First, fungal EVs can contain virulence-associated molecules, including GXM in *Cryptococcus* and Bgl2p and Hgt1p in *Candida*. Other reported components include polysaccharides, lipids, and small non-coding RNAs, with cargo composition varying among species and experimental conditions. Second, fungal EVs can modulate macrophage polarization and cytokine production, thereby shaping host immune responses. Third, they may contribute to antifungal resistance and biofilm-associated persistence. Finally, fungal EVs appear to participate in fungal adaptation to environmental and host-derived stress conditions. These findings indicate that fungal EVs should not be regarded as a uniform functional entity. Rather, they represent species-specific, dynamic communication platforms whose cargo and biological effects vary with pathogenic strategy and host environment, contributing to diverse aspects of host–pathogen interactions.

### Prospects for the application of EVs in the diagnosis and treatment of fungal infections

EVs are broadly distributed across different cell types and can be released into a variety of extracellular fluids, including blood, urine, saliva, and other biological fluids (Merchant et al., 2017; Todorova et al., 2017). They carry diverse biomolecules, such as proteins, nucleic acids, lipids, and metabolites, many of which can reflect the physiological or pathological status of their cells of origin (Van Niel et al., 2018; Tian et al., 2023). By transferring these bioactive molecules to neighboring or distant recipient cells, EVs facilitate intercellular communication and regulate a wide range of biological processes (Van Niel et al., 2018; Möller and Lobb, 2020). These properties have attracted considerable interest in EVs as potential diagnostic biomarkers, therapeutic targets, and drug-delivery systems (Wiklander et al., 2019; Dallastella et al., 2023). The 2013 Nobel Prize in Physiology or Medicine recognized the importance of vesicular trafficking in cell biology, awarded to James E. Rothman, Randy W. Schekman, and Thomas C. Südhof for their discoveries of the molecular machinery that regulates vesicle trafficking and membrane fusion. Their work demonstrated that conserved mechanisms of vesicular transport operate across eukaryotic organisms, from yeast to humans, providing fundamental insights into cellular organization and intercellular communication. These discoveries have expanded our understanding of the mechanisms underlying disease pathogenesis and provided a basis for identifying potential therapeutic targets (Todorova et al., 2017; Dallastella et al., 2023). Since then, research into EVs has expanded rapidly over the past decade, accompanied by the emergence of several innovative companies focused on EV-related research and applications, including Codiak BioSciences, Exosome Diagnostics, and Exosome Sciences. Although research on fungal EVs remains in its early stages, their potential value in elucidating fungal pathophysiology, the development and progression of fungal infections, and the regulation of host immune responses is increasingly recognized (Li et al., 2026b). As fundamental research advances, the potential applications of fungal EVs as biomarkers for the diagnosis and prognosis of fungal infections, as well as drug-delivery vehicles or vaccine platforms for the targeted prevention and treatment of IFIs, are expected to continue to expand.

### Diagnostic marker for fungal infections

Numerous studies have demonstrated the potential of EVs for the early screening and laboratory diagnosis of various diseases, including cancer (Möller and Lobb, 2020), cardiovascular diseases (Jansen et al., 2017), renal diseases (Erdbrügger and Le, 2015), and neurodegenerative diseases (Thompson et al., 2016). Compared with conventional approaches such as tissue biopsy, EV-based liquid biopsy offers a less invasive and more accessible diagnostic approach while providing molecular information that may reflect disease heterogeneity and progression (Kim et al., 2017). Fungal EVs are present in various biological fluids and contain biomolecules derived from their cells of origin. Their abundance, molecular composition, and cargo can vary according to physiological conditions, environmental stimuli, and disease states, providing a potential basis for their use as diagnostic and prognostic biomarkers (Albuquerque et al., 2008; Gandhi et al., 2022; Rizzo et al., 2023; Yuan et al., 2024; de Rezende et al., 2025; Lee et al., 2025). With advances in EV isolation, molecular profiling, and detection technologies, fungal EVs may provide new opportunities for the early diagnosis and prognosis of fungal infections (Rodrigues et al., 2014; Souza et al., 2019).

Despite the promising diagnostic potential of fungal EVs, several technical and clinical limitations currently hinder their routine clinical application. First, the sensitivity and specificity of EV-based fungal diagnostics remain highly dependent on sample source, fungal burden, EV isolation methods, and detection platforms (Baltazar et al., 2018; Halder et al., 2022). As fungal EVs are often present at relatively low abundance in clinical specimens, especially during early-stage infection, reliable identification requires efficient enrichment and highly sensitive detection technologies (Bielska et al., 2018; Ullah et al., 2023; de Rezende et al., 2025).

A major challenge is the lack of standardized protocols for EV isolation, purification, quantification, and characterization. Current approaches include differential ultracentrifugation, density-gradient centrifugation, size-exclusion chromatography, precipitation-based methods, and immunoaffinity capture. These techniques differ in recovery efficiency, purity, scalability, cost, and potential effects on EV integrity and cargo composition (Panepinto et al., 2009; Silva et al., 2013; Vargas et al., 2015; de Paula et al., 2019). In addition, contamination with host-derived EVs, serum proteins, lipoproteins, and cellular debris remains a major challenge for detecting fungal EV-specific biomarkers (Oliveira et al., 2010a; Silva et al., 2013; Vargas et al., 2015; de Paula et al., 2019). de Rezende et al. report the concurrent presence of host and fungal EVs, highlighting that this issue remains unresolved (de Rezende et al., 2025). The standard fluid isolation techniques co-purify host vesicles, and current methodologies such as particle tracking and electron microscopy are unable to differentiate their origins, indicating that these technical challenges persist. Furthermore, it should be explicitly noted that existing human data are limited to a small number of pathogens and studies, and a clinical method for distinguishing fungal EVs from host EVs has yet to be developed. In relation to comparisons with antigen tests currently in clinical use, such as galactomannan, beta-D-glucan, and cryptococcal antigen, we cautiously note that benchmarking against these standards has not yet been conducted.

From a translational perspective, most fungal EV-based diagnostic strategies remain at the experimental or preclinical stage (Bitencourt et al., 2018; Rizzo et al., 2023; Yuan et al., 2024; de Rezende et al., 2025; Lee et al., 2025). Large, multicenter clinical studies evaluating diagnostic sensitivity, specificity, positive predictive value, and prognostic performance in patients with invasive fungal infections are still limited. In addition, routine hospital laboratories still need rapid, cost-effective, and clinically adaptable detection platforms. Emerging technologies, including microfluidics, nanosensors, single-vesicle analysis, and integrated multi-omics profiling, may improve the sensitivity, specificity, and clinical feasibility of fungal EV-based diagnostic assays (Kwaku et al., 2024; Shi et al., 2025; Pawar et al., 2026).

Beyond naturally occurring fungal EVs, synthetic and semisynthetic vesicular systems have emerged as potential tools for studying fungal biology and developing antifungal therapies. Fungal synthetic vesicles are nano-sized membranous carriers constructed *in vitro* from purified lipids, proteins, or other biomaterials, with liposomes representing a common example. Researchers can precisely control their composition, size, surface charge, and encapsulated cargo (Aguilar-Pérez et al., 2021). Semisynthetic vesicles use naturally derived vesicles as scaffolds and acquire additional functions through approaches such as chemical conjugation, genetic engineering, or membrane fusion, thereby combining features of biological vesicles with the controllability of engineered systems (Piraine et al., 2026). In basic research, synthetic and semisynthetic vesicles have been used to mimic fungal membrane properties, lipid raft microdomains, and mechanisms involved in host-cell recognition and endocytosis (Puerner et al., 2021; Tevyashova et al., 2023; Sarkar et al., 2026). These engineered vesicular systems also have potential biomedical applications. Liposomes, ethosomes, and transfersomes can encapsulate antifungal agents such as natamycin, ketoconazole, and luliconazole, potentially improving their solubility, stability, and tissue penetration while enabling targeted delivery through surface modification (Ahmed et al., 2021; Nagasa and Belete, 2022; Singh et al., 2025). Components involved in vesicle trafficking may also provide potential targets for the development of antibiofilm and anti-infective strategies (Zhao et al., 2021; Zhang et al., 2022). In biotechnology, fungal membrane proteins can be reconstituted into synthetic vesicles to develop biosensors, while vesicular systems can serve as carriers for enzyme immobilization and catalytic reactions (Antoniel et al., 2026). Compared with some conventional synthetic nanocarriers, fungal-derived and biomimetic vesicles may offer advantages related to biocompatibility, membrane composition, and interaction with target cells. Surface engineering can also modify their physicochemical and biological properties (Tevyashova et al., 2023; Sarkar et al., 2026). However, challenges remain, including limited scalability, batch-to-batch variability, insufficient characterization of *in vivo* behavior and immunogenicity, lack of standardized evaluation methods, and uncertainty regarding cost-effectiveness and clinical translation (Rebollo et al., 2022; Chen et al., 2023).

Standardized methodological frameworks will therefore be critical for advancing research on fungal EVs. The International Society for Extracellular Vesicles (ISEV) has established the Minimal Information for Studies of Extracellular Vesicles (MISEV) guidelines, which recommend EV nomenclature, isolation, characterization, experimental controls, and functional validation (Merchant et al., 2017). Although these guidelines were developed primarily from mammalian EV research, they offer a valuable framework for improving methodological rigor and consistency in reporting fungal EV studies. However, directly applying MISEV recommendations to fungal systems remains challenging because fungal EVs possess distinct structural and biological features, including a rigid cell wall, highly heterogeneous cargo, and species-specific mechanisms of vesicle formation and secretion (Gandhi et al., 2022; Jnana et al., 2023; Ullah et al., 2023; Lee et al., 2025). Candidate fungal EV markers have already been identified, including the claudin-like proteins Sur7 and Evp1 in *C. albicans (*Dawson et al., 2020). More recently, ergosterol was detected in serum EV preparations from patients with candidiasis, cryptococcosis, and paracoccidioidomycosis, suggesting its potential as an infection-associated biomarker (de Rezende et al., 2025). Accordingly, fungal EV research requires optimized isolation workflows, further validation of existing candidate markers and assessment of their cross-species applicability, standardized quantification methods, and harmonized reporting criteria. Establishing such frameworks will enhance reproducibility and facilitate the translation of fungal EV research from experimental settings into clinical applications.

### As anti-fungal drug delivery vehicles

EV-based drug delivery has emerged as a promising strategy for transporting chemical or genetic therapeutics. EVs can encapsulate nucleic acids, peptides, lipids, and small-molecule compounds and may improve the solubility, stability, bioavailability, and cellular delivery of therapeutic agents while reducing systemic toxicity (Kim et al., 2017; Armstrong and Stevens, 2018; Timin et al., 2018). Researchers have also investigated bacterial EVs as delivery vehicles for functional nucleic acids, peptides, lipids, and small-molecule drugs, with their biological origin and surface properties potentially affecting tissue distribution and cellular uptake (Kim et al., 2017). Compared with bacterial and mammalian EV-based delivery systems, the development of fungal EVs as carriers for antifungal therapeutics remains in the early stages. However, their native membrane composition, cargo-loading capacity, and ability to interact with both fungal and host cells provide a foundation for further exploration (Zarnowski et al., 2021; Bitencourt et al., 2022). Fungal EVs could potentially be engineered to deliver antifungal compounds or nucleic acid-based therapeutics directly to fungal cells or infected tissues, thereby enhancing local drug exposure while minimizing off-target effects (Kim et al., 2017; Sarkar et al., 2026). Future studies should therefore investigate cargo-loading efficiency, vesicle stability, biodistribution, cellular targeting, pharmacokinetics, toxicity, and the potential impact of fungal EV components on host immune responses.

### Potential fungal vaccine

The increasing incidence of invasive fungal infections, emergence of antifungal resistance, and limited antifungal therapeutic options have increased the need for preventive strategies. Despite significant progress in fungal vaccine research, no fungal vaccine is currently approved for human clinical use. Fungal EVs are attractive vaccine candidates because they contain multiple fungal antigens and immunomodulatory molecules that can stimulate innate and adaptive immune responses. EVs derived from *C. albicans*, *C. neoformans*, and *A. flavus* have been reported to protect *G. mellonella* against fungal infection in a dose-dependent manner, at least in part by activating innate immune responses (Vargas et al., 2015; Colombo et al., 2019; Brauer et al., 2020). In Balb/c mice, immunization with EVs enriched in the *C. albicans* immunogenic protein Bgl2p provided partial protection against *C. albicans* infection. Moreover, sera from patients with candidiasis recognize antigens present in *C. albicans* EVs, indicating that these vesicles contain clinically relevant immunogenic components (Gil-Bona et al., 2015). Similarly, sera from patients with paracoccidioidomycosis recognize *P. brasiliensis* EVs, further supporting their potential as sources of protective antigens (Vallejo et al., 2011).

Although existing studies of fungal EVs are highly heterogeneous in experimental design, fungal species, EV isolation methods, host models, and analytical platforms, several consistent patterns have emerged across the literature (Baltazar et al., 2018; Campos et al., 2021; Yang et al., 2021; Souza et al., 2022). Comparative synthesis of the available evidence indicates that fungal EVs can regulate innate immune responses by modulating macrophage activation, cytokine production, neutrophil recruitment, inflammasome signaling, and biofilm-associated antifungal resistance (Reis et al., 2021; Freitas et al., 2023). Importantly, direct quantitative meta-analysis remains challenging because of substantial inter-study variability in EV purification methods, particle quantification approaches, fungal culture conditions, host models, and outcome measures (Wolf et al., 2015; Castelli et al., 2022; Souza et al., 2022). Therefore, this review emphasizes qualitative comparative synthesis and mechanistic integration as more appropriate approaches at the current stage of fungal EV research. Further standardization of experimental methodologies and the development of large-scale datasets may ultimately enable more robust quantitative meta-analyses (**Table 1**). Due to the lack of standardized criteria for EV isolation methods and doses, preparation trends are for reference only and not for direct comparison.

**Table 1:** The effects of fungal EVs on the host immune system.

EVs from	Host	Pro-inflammatory cytokines								Anti-inflammatory cytokines				PI	Ref.
		IFN-γ	TNF-α	IL-1α	IL-1β	IL-6	IL-8	IL-12	NO	TGFβ	IL-4	IL-10	IL-13		
*Cryptococcus neoformans*	Murine macrophages RAW 264.7		↑						↑	↑		↑		↑	(Oliveira et al., 2010b)
*Cryptococcus neoformans*	In vivo Acanthamoeba castellanii model of infection													≌	(Rizzo et al., 2017)
*Cryptococcus gattii*	Murine macrophages J774A.1													≌	(Bielska et al., 2018)
*Malassezia sympodialis*	Human PBMCs(healthy donors)/CD14,CD34 depleted PBMCs(healthy or AE donors)		↑								↑				(Gehrmann et al., 2011)
*Trichophyton interdigitale*	Murine Bone marrow-derived macrophages (Murine BMDMs)		↑		↑	↑			↑				≌	↑	(Bitencourt et al., 2018)
*Trichophyton interdigitale*	Human keratinocytes(HaCaT)		↑		↑	↑	↑		↑						(Bitencourt et al., 2018)
*Sporothrix brasiliensis*	Mice BMDDCs	↑	↑		≌	≌		↑			≌	≌		↑	(Ikeda et al., 2018)
*Sporothrix brasiliensis*	In vivo murine skin model of infection		↑		↑										(Ikeda et al., 2018)
*Candida albicans*	Murine macrophages RAW 267.4/murine BMDMs/murine BMDDCs		↑					↑		↑		↑			(Vargas et al., 2015)
*Histoplasma capsulatum*	Murine BMDMs													↓	(Baltazar et al., 2018)
*Histoplasma capsulatum*	Human macrophages (THP-1-derived macrophages)													≌	(Baltazar et al., 2018)
*Cryptococcus neoformans(virulent capsular strain/hypocapsular avirulent mutant)*	Mice BMDMs/Mice BMDCs		↑		↑										(Marina et al., 2020)
*Cryptococcus neoformans(virulent capsular strain/hypocapsular avirulent mutant)*	In vivo C57BL/6 mice model of infection		↑		↑	↑									(Marina et al., 2020)
*Candida albicans*	In vivo BALB/c mice model of infection	↑	↑			↑		↑		↑	↑	↑			(Vargas et al., 2020)
*Candida albicans*	Mice BMDCs					↑				↑				≌	(Zamith-Miranda et al., 2021)
*Candida auris*	Mice BMDCs					↑				↑				≌	(Zamith-Miranda et al., 2021)
*Candida glabrata*	THP-1 cells		↑				↑					↑			(Kulig et al., 2022)
*Candida parapsilosis*	THP-1 cells		↑				↑					↑			(Kulig et al., 2022)
*Candida tropicalis*	THP-1 cells		↑				↑					↓			(Kulig et al., 2022)
*Candida albicans(hyphal cells / yeast )*	THP-1 cells		↑												(Martínez-López et al., 2022)
*Aspergillus fumigatus*	Murine macrophages RAW 264.7		↑											↑	(Souza et al., 2019)
*Aspergillus fumigatus*	Murine bone marrow-derived neutrophils (BMDNs)		↑		↑									↑	(Souza et al., 2019)
*Aspergillus flavus*	Murine BMDMs		↑		↑	↑			↑					↑	(Brauer et al., 2020)
*Aspergillus fumigatus*	Male C57BL/6 mice				↓	↓								↑	(Souza et al., 2022)
*Paracoccidioides brasiliensis*	Male C57BL/6 mice	↑													(Baltazar et al., 2021)
*Paracoccidioides brasiliensis*	Murine macrophages RAW 264.7		↑			↑			↑						(Da Silva et al., 2016)
*Sporothrix brasiliensis*	Macrophage J774					↑		↑						↓	(Campos et al., 2021)

EVs, extracellular vesicles; BMDM, bone-marrow-derived macrophages; BMDC, bone-marrow-derived dendritic cells; BMDN, bone-marrow-derived neutrophils; PBMC, peripheral-blood mononuclear cells; PI, phagocytic index; ↑, increased; ↓, decreased; ≌, no significant change. Due to the lack of standardized criteria for EV isolation methods and doses, preparation trends are for reference only and not for direct comparison.

## Conclusion

Fungal EVs have emerged as important mediators of intercellular communication, fungal virulence, immune modulation, biofilm development, and host–pathogen interactions. Evidence from pathogenic fungi indicates that EVs are dynamic biological structures whose abundance, composition, and activity can vary in response to environmental stress, antifungal exposure, fungal morphogenesis, and host immune pressure. By selectively transporting proteins, lipids, polysaccharides, nucleic acids, and other bioactive molecules, fungal EVs can influence both fungal physiology and host immune responses. EVs are promising candidates for use as biomarkers, therapeutic targets, drug-delivery systems, and vaccine platforms for invasive fungal infections. Despite these advances, however, fundamental aspects of fungal EV biology remain poorly understood. The molecular mechanisms governing EV biogenesis, cargo selection, secretion, and passage through the fungal cell wall require further investigation, particularly regarding the respective contributions and coordination of ESCRT-dependent and ESCRT-independent pathways. Although candidate EV markers such as Sur7 and Evp1 have been reported in *C. albicans (*Dawson et al., 2020), their reliability across diverse fungal species and experimental conditions remains to be established. The marked heterogeneity of fungal EV populations further underscores the need for fungal-specific standards governing EV isolation, purification, quantification, characterization, and functional assessment. Standardized experimental protocols and reporting criteria will be essential to improve reproducibility and enable meaningful comparisons across studies.

Future research should clarify how fungal species, environmental conditions, antifungal treatment, and morphological transitions influence EV heterogeneity and biological activity. Integrating single-vesicle analysis, high-resolution imaging, multi-omics profiling, live-cell tracking, and computational approaches may provide a more comprehensive understanding of EV-mediated communication and cargo selection.

From a translational perspective, well-designed multicenter clinical studies are needed to establish the diagnostic and prognostic value of fungal EV-associated biomarkers in invasive fungal infections. In addition, engineering fungal or fungal-inspired vesicles for targeted drug delivery and vaccination represents a promising but still experimental strategy that requires rigorous evaluation of efficacy, safety, pharmacokinetics, and immunogenicity. Overall, fungal EV research is advancing from descriptive characterization toward mechanistic and translational investigation. Continued integration of fungal biology, immunology, nanotechnology, omics technologies, and clinical research will be critical for determining whether the unique biological properties of fungal EVs can ultimately be translated into reliable diagnostic tools and effective therapeutic or preventive strategies for invasive fungal infections.
